# STK31 enhances gallbladder cancer development by modulating DNA replication machinery

**DOI:** 10.1016/j.jbc.2026.113534

**Published:** 2026-09-08

**Authors:** Bin Zhang, Hai-Ying Peng, Zhu-Ying Lin, Wei-Yu Zhu, Shuang-Xin Li, Song-Lin Yang, Qiu-Wen Ye, Yi-Yi Zhao, Hui Gao, Shan He, Yuan Liu, Xiao-Dan Wang, Yang-Fan Guo, Wen-Ju Wang, Li-Wei Liao, Zong-Liu Hou, Ming-Yao Meng, Lin Li

**Affiliations:** 1 Central Laboratory of Yan’an Hospital Affiliated to Kunming Medical University, Kunming, Yunnan Province, China; 2 Key Laboratory of Tumor Immunological Prevention and Treatment of Yunnan Province, Kunming, Yunnan Province, China; 3 Kunming Medical University, Kunming, Yunnan Province, China; 4 The Third Hospital Affiliated to Kunming Medical University, Kunming, Yunnan Province, China

**Keywords:** Atovaquone, c-Myc, DNA replication, gallbladder cancer, MCMs, STK31

## Abstract

Gallbladder cancer (GBC) is a highly aggressive and insidious malignancy with a poor prognosis, primarily due to the lack of specific therapeutic targets and early diagnostic biomarkers. Therefore, identifying novel oncogenes involved in GBC and exploring targeted therapeutic strategies against them are of particular importance. STK31 is a cancer/testis antigen, but its biological function and mechanistic role in GBC remain unclear. This study investigates how STK31 influences GBC. STK31 expression in GBC and gastrointestinal tumors were analyzed using collected clinical samples and TCGA database, respectively. Its pro-tumorigenic functions were then confirmed by *in vivo* and *in vitro* assays, while downstream regulatory mechanisms were uncovered *via* multi-platform proteomic strategies. Molecular docking was employed to screen 1008 ChEMBL small molecules post STK31 functional validation. Significant upregulation of STK31 expression was observed in both GBC and gastrointestinal malignancies through our comprehensive analysis. STK31 depletion decreased GBC cell proliferation, migration, and induced G1/S phase arrest and cellular polyploidization. Gene Set Enrichment Analysis (GSEA) demonstrated that STK31 functionally correlates with core biological processes including DNA Replication, Cell Cycle, and Extracellular Matrix (ECM) Receptor Interaction. STK31 directly binds to both c-Myc and FOXP1, thereby regulating MCM and ITG family proteins to exert its biological function. In addition, STK31 influences the phosphorylation of c-Myc through its kinase activity. Atovaquone identified through molecular docking screening, binds STK31 and reduces its expression and kinase activity, demonstrating anti-tumor effects against GBC both *in vitro* and *in vivo*. We demonstrate that STK31 facilitates GBC progression through c-Myc/FOXP1-mediated DNA replication dysregulation. Its inhibition triggers lethal polyploidization, with ATO identified as a potential STK31-targeting drug candidate.

Gallbladder cancer (GBC) is a malignant tumor of the biliary tract that arises from the epithelial cells of the gallbladder mucosa and the cystic duct, with adenocarcinoma being the predominant histological type. It is a rare but highly aggressive malignancy, and the majority of cases are diagnosed at an advanced stage due to the absence of early symptoms (1). The incidence rate of GBC is the sixth in the malignant tumors of digestive system. Furthermore, GBC shows poor responsiveness to conventional radiotherapy and chemotherapy, contributing to its dismal prognosis (2). Identifying gallbladder cancer-specific oncogenes and elucidating their molecular mechanisms represent critical breakthroughs in the clinical research of GBC. Such discoveries are expected to provide novel strategies for targeted therapeutic interventions and early detection, ultimately improving patient outcomes (3).

Serine/threonine kinase 31 (STK31) is a member of the serine/threonine kinase family, which regulates various cellular processes through phosphorylation. STK31 is involved in the regulation of several key cellular processes, including cell proliferation, cell cycle progression, spindle assembly, and chromosome stability (4, 5). STK31 has recently been identified as a novel cancer/testis antigen (CTA), characterized by its highly restricted expression pattern in normal human tissues—being predominantly expressed in the testis. Some previous studies have shown that STK31 is aberrantly expressed in various malignancies including lung cancer, colorectal cancer, pancreatic cancer, gastric cancer, esophageal cancer, and so on (6, 7, 8, 9). However, to date, there have been no studies investigating the role of STK31 in GBC. Moreover, the precise molecular mechanisms by which STK31 contributes to GBC development and progression remain largely unclear, and further investigation is warranted to elucidate its biological functions and therapeutic implications. As a CT antigen, STK31 not only represents a promising biomarker for cancer diagnosis and prognosis but also serves as a potential target for immunotherapy and other precision medicine strategies.

Atovaquone (ATO) is a hydroxy-1,4-naphthoquinone compound initially developed for the treatment and prevention of protozoan infections. Its primary mechanism of action involves the inhibition of the cytochrome-bc1 complex in the mitochondrial electron transport chain of the pathogen, thereby blocking electron transfer and ATP synthesis, ultimately leading to pathogen death (10). In recent years, emerging studies have revealed the potential of ATO in the field of cancer therapy (11, 12, 13).

In this study, the key genes that enhance the development of GBC would be identified by using microarray analysis of GBC clinical specimens, and STK31 was strongly expressed in GBC. Furthermore, STK31 depletion alleviated GBC proliferation and metastasis through increasing the expression of MCM family proteins while suppressing the expression of integrin (ITG) family proteins. STK31 may contribute to the polyploidization of GBC cells by enhancing c-Myc and FOXP1 expression. STK31 increased c-Myc expression by affecting its phosphorylation through its kinase activity. Through molecular docking analysis, we identified a potential interaction between ATO and STK31. Subsequent experiments confirmed the binding and the functional roles of ATO in GBC *in vitro* and *in vivo*. Our findings uncover the role of STK31 in GBC by altering DNA replication machinery, proposing STK31 as a potential therapeutic target for GBC therapy.

## Results

### STK31 is a potential carcinogenesis-related gene

In order to identify differentially expressed genes (DEGs) in GBC, tissue microarray analyses (TMA) for five pairs of GBC and normal paracarcinoma tissues were conducted. In total, 393 DEGs were screened, among which 262 genes including STK31 were significantly upregulated (log2(fold change)∣ ≥ 1, *p* value < 0.05) (Fig. 1*A*). In addition, higher STK31 expression was associated with poorer overall survival (OS) outcomes in both pan-cancer and digestive system cancer cohorts from TCGA database (Fig. 1*B*). Previous studies have also found that STK31 is responsible for tumor formation and metastasis, especially in digestive system tumors such as colorectal cancer, pancreatic cancer, gastric cancer, esophageal cancer, etc (4, 7, 8, 9, 14). The results demonstrated that STK31 expression was significantly upregulated in multiple digestive system cancer tissues compared to their normal counterparts from GEPIA2 bioinformatics analysis (http://gepia.cancer-pku.cn, Fig. 1*C*). Therefore, the expression of STK31 was validated by real-time PCR in tissue from 15 cases of GBC and normal paracarcinoma tissues. The result showed that *STK31* gene expressions in GBC tissue were significantly higher than those in normal paracarcinoma tissues (Fig. 1*D*). Meanwhile, immunohistochemical detection of STK31 expression in GBC (n = 72) and chronic cholecystitis tissues (n = 108) demonstrated that STK31 expression was markedly increased in GBC tissues compared to cholecystitis (*p* < 0.001, Fig. 1*E*). According to the Human Protein Atlas database, STK31 exhibits enhanced RNA expression levels in gallbladder cancer and gastric cancer cell lines (https://www.proteinatlas.org/ENSG00000196335-STK31/cell+line). We also evaluated the expression of STK31 protein between GBC cell lines (GBC-SD, NOZ) and normal epithelial cells in gallbladder mucosa (Fig. S1*A*) by Western blot. The data showed that STK31 was highly expressed in GBC cell lines, however not expressed in normal epithelial cells (Fig. 1*F*). Additionally, we collected adjacent non-cancerous tissues and cancerous tissues and examined STK31 expression using immunohistochemistry (6 pairs of samples) and Western blot (1 pair of samples). The results showed that STK31 expression was indeed increased in cancerous tissues compared with adjacent non-cancerous tissues (Fig. S1, *B* and *C*). These results illustrated that STK31 is an important carcinogenic factor in GBC.

**Figure 1:**
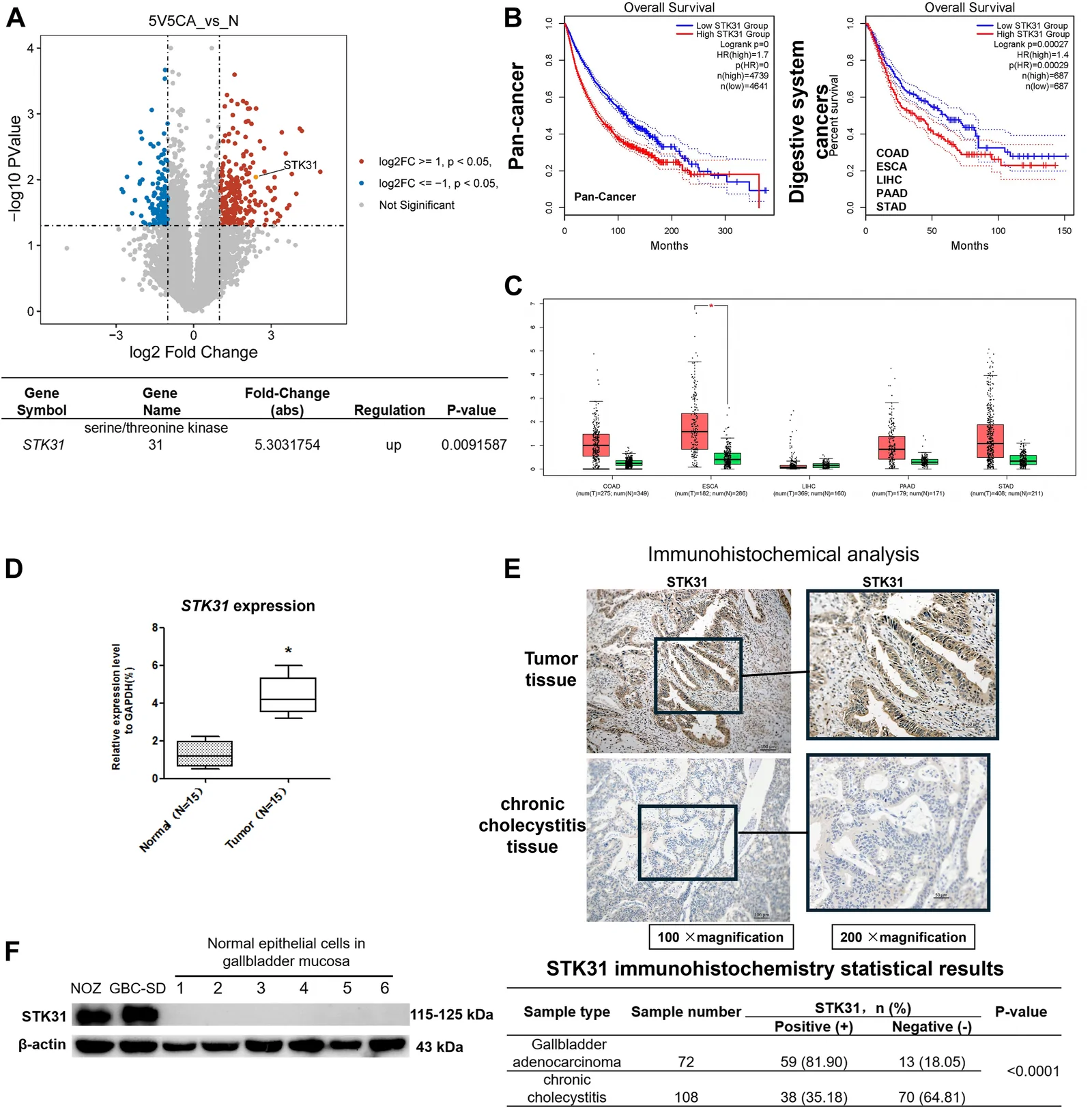
**STK31 as a potential carcinogenesis-related gene was significantly upregulated in GBC tissues**. *A*, Volcano plot showing 393 differentially expressed genes (DEGs,∣log2(fold change)∣ ≥ 1, *p* value < 0.05) between GBC and normal paracarcinoma tissues. Among them, 262 upregulated (*red*) genes in GBC including STK31. *B*, based on TCGA database, patients with higher STK31 expression showed lower overall survival (OS) compared to patients with low STK31 expression in pan-cancer and digestive system cancers (colon adenocarcinoma, COAD; esophageal carcinoma, ESCA; liver hepatocellular carcinoma, LIHC; pancreatic adenocarcinoma, PAAD; stomach adenocarcinoma, STAD) (log-rank test, two-sided, *p* = 0.00027). The survival analysis and Kaplan-Meier curve were performed and plotted through GEPIA 2 (http://gepia2.cancer-pku.cn/#survival). *C*, the expression patterns of STK31 across above digestive system cancers and their paired normal tissues were also characterized through GEPIA2 bioinformatics analysis. *Red bars*: tumor tissues; green bars: matched normal tissues. *D*, STK31 gene expression in tumor and normal tissue was validated by RT-PCR (n = 15). *E*, representative images of GB adenocarcinoma specimens (n = 72) and chronic cholecystitis (n = 108) showing positive and negative staining of STK31 in sections after IHC staining and STK31 IHC statistical results. The difference between the two groups was determined by chi-square test, *p* < 0.0001. *F*, STK31 expressions of normal epithelial cells from gallbladder mucosa after primary culture and GB cancer cell lines GBC-SD and NOZ were detected by Western blot. Actin expression was determined to confirm equal protein loading.

### STK31 depletion suppressed GBC proliferation, colony formation, and migration while inducing cell cycle arrest

Given STK31 plays a potential vital role in gallbladder tumorigenesis, its impacts on the abilities of proliferation and migration on GBC cells were evaluated with CCK-8, colony formation and transwell assays, respectively. By using lentivirus transfection (15), the expression of STK31 was significantly reduced in GBC-SD and NOZ cells, especially shSTK31-05 and shSTK31-06 interference sequences (Fig. 2*A*, Fig. S2, *A* and *B*). Meanwhile, cell proliferation was remarkably hindered after STK31 knockdown (Fig. 2*B*). The cell cycle analysis showed that the G1 phase percentage increased in shSTK31-expressing cells with an obvious reduction in S phase, suggesting G1 arrest (Fig. 2*C*). Further experiments showed impaired clone formation abilities in STK31 knockdown cells (Fig. 2, *D*–*F*). To assess cell migration abilities, Boyden chamber assay was performed after STK31 knockdown. Low STK31 expression cells showed significantly reduced migration tendency (Fig. 2, *G*–*I*).

**Figure 2:**
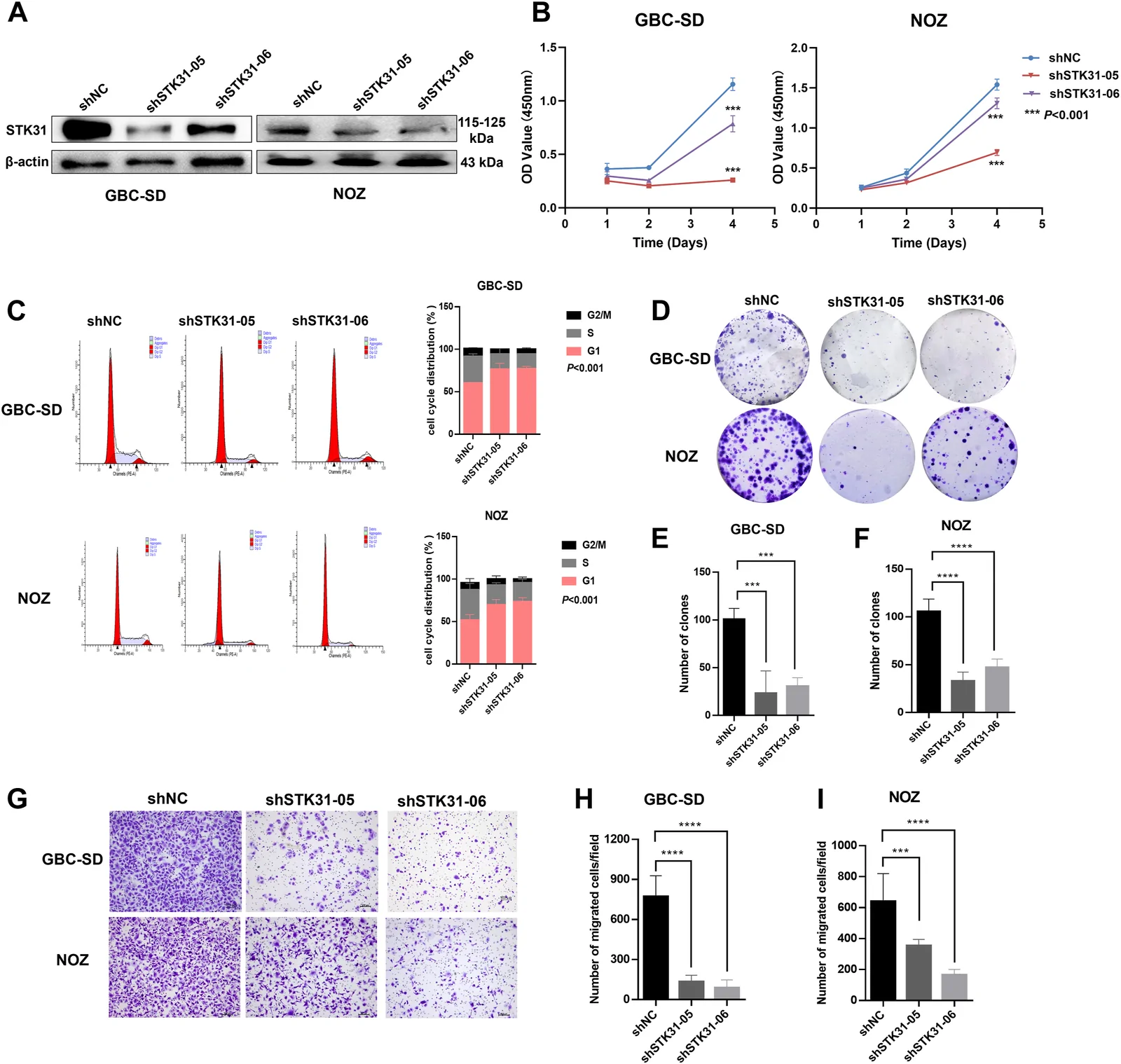
**STK31 depletion suppressed GBC-SD and NOZ cells proliferation, clone formation, migration and induced cell cycle arrest**. *A*, representative immunoblots showed the effects of knock-down on the STK31 protein expression of GBC-SD and NOZ cells. STK31 expression after lentiviral transfection was used to verify by Western blot, which was only significantly reduced in shSTK31-05 and shSTK31-06 expressing cells. *B*, CCK-8 assays were performed to measure cell proliferation. Data were expressed as means ± SD from three independent experiments. *C*, representative diagrams of cell cycle evaluated by flow cytometry. The cell cycle distributions of each phase of GC cells are analyzed in bar chart with percentage. The data was shown as mean ± SD of three independent experiments. Chi-square test is used to compare the distribution differences of cell cycle between shNC control group and shSTK31 groups, ∗∗∗*p* < 0.001. *D*, representative photos showing the stained cells on the clone formation assay. Cells were seeded in 6-well plates for 10 or 12 days, the clones were fixed and stained for counting. *E* and *F*, quantification of counted clones. Results are expressed as the number of clones, which are from three independent experiments. *G*, representative photos showing the stained cells on the lower side of the transwell plate membrane. The above cells migrated to the lower chambers were stained and the numbers were counted. *H* and *I*, quantification of migration of GBC-SD and NOZ cells. Results are expressed as the mean cell numbers (mean ± SD of three independent experiments). Differences between shNC group and shSTK31 groups were determined by one-way ANOVA followed by *post hoc* Dunnett’s test, ∗∗∗*p* < 0.001, ∗∗∗∗*p* < 0.0001.

### The proteins involved in DNA replication, cell cycle, and ECM receptor interaction responsible for STK31 downstream function

In order to determine the proteins responsible for STK31 downstream functional mechanism, we conducted 4D-lable free proteomic on proteins from STK31 knockdown cells and analyzed the relationship between different protein expressions, biological processes, cellular components affected, and molecular functions involved in KEGG using GSEA. We applied stringent enrichment screening criteria, defining significance as *p* < 0.05 and visualized the data through ridge plots and pathway maps in KEGG. Among the top 20 shown, our results revealed a notable enrichment of genes in pathways such as DNA replication, cell cycle, and ECM receptor interaction (Fig. 3, *A*–*C*). Notably, proteins linked to DNA Replication and cell cycle displayed marked upregulation in cells from shSTK31 group compared with control group, while proteins associated with ECR Receptor Interaction showed obvious downregulation (Fig. 3, *B* and *C*). In addition, the changes of some proteins including Minichromosome Maintenance proteins (MCMs) and Integrins (ITGs) family from the results of 4D-lable proteomics were validated by LC-MS, which were consistent with the proteomics findings finally (Fig. 3*D*). Furthermore, CDC7, a serine-threonine protein kinase, also promotes DNA initiation and replication by activating the MCM complex, ensuring smooth DNA replication during the cell cycle (16, 17). To elucidate the interaction of differentially expressed proteins of cells involved in DNA Replication, Cell Cycle and ECM Receptor Interaction pathways and the molecules it regulated, the target proteins including CDC7 were analyzed by STRING database, which provided associations and links between query genes STK31 and the above proteins. Figure 3*E* showed the connectivity network of proteins with differential expression in GBC-SD cells after knockdown of STK31.

**Figure 3:**
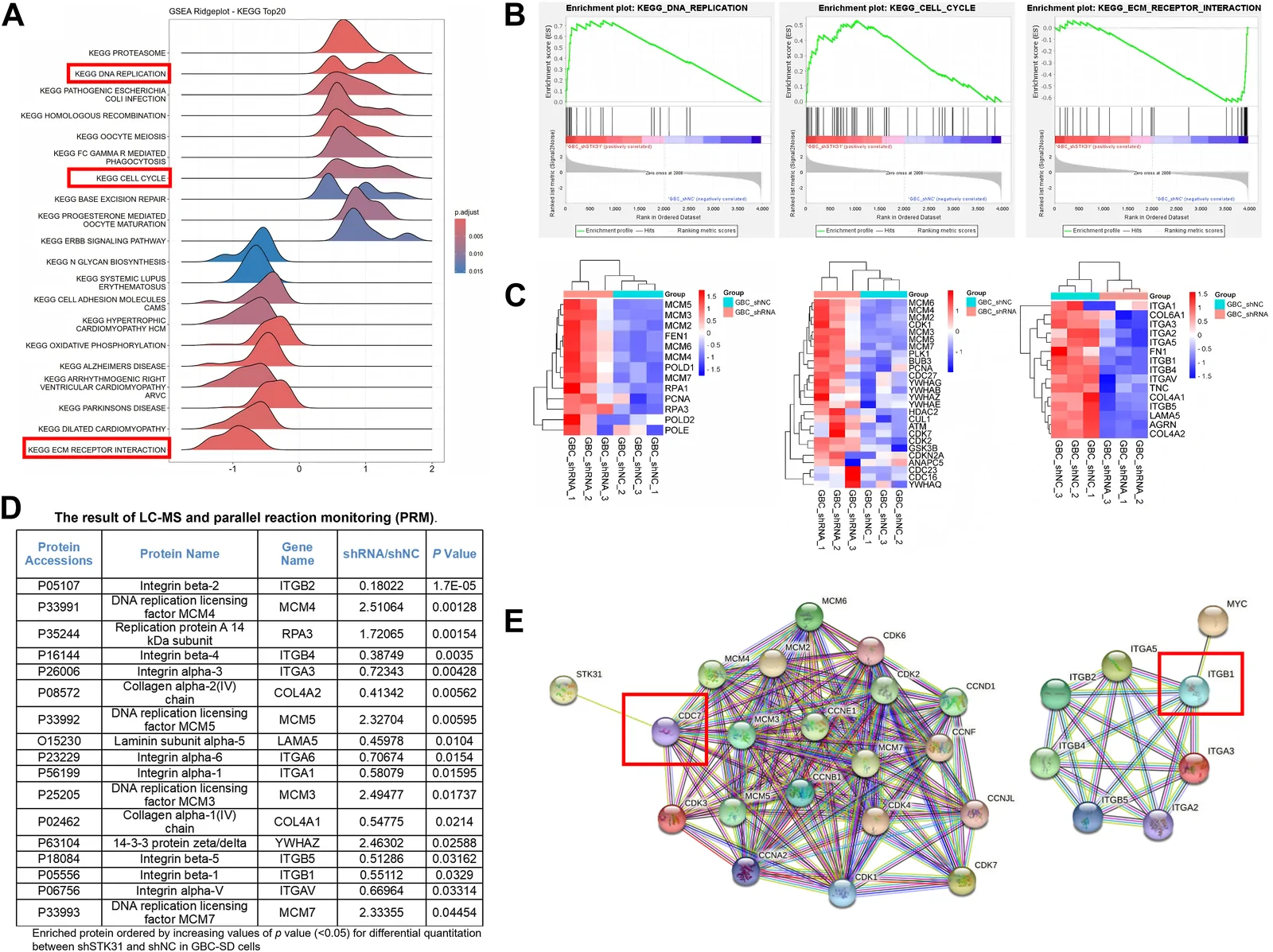
**Bioinformatic analysis of proteomics data after knock-down of STK31 in GBC-SD**. *A*, a ridgeplot of GSEA (Gene set enrichment analysis) with gene sets from biological function and pathway using KEGG classification, and the *top* 20 were shown. The numbers at the *bottom* of the plot represent Normalized Enrichment Score, NES. The position of the peak indicates whether the enriched differentially expressed proteins (DEPs) in this pathway are up-regulated (NES > 0) or down-regulated (NES < 0) compared with shNC, and the height of the peak represents the number of enriched DEPs. *B*, GSEA of proteins signatures of DNA Replication, Cell Cycle and ECM Receptor Interaction pathway in shSTK31 knock-down GBC-SD cells. *C*, heatmap showing the significantly enriched proteins of DNA Replication, Cell Cycle and ECM Receptor Interaction pathway from GSEA analysis (total 48 proteins, *p* < 0.05). *D*, the changes of some proteins from the results of 4D-lable proteomics were validated by LC-MS (*p* < 0.05). *E*, protein–protein interaction analysis by STRING database. The line width represented the strength of the interaction between two proteins.

### STK31 depletion increased the expression of MCM family proteins while suppressing the expression of ITG family proteins

The MCM family mainly includes six core proteins, MCM2-MCM7, forming a hexameric complex that is a key component for initiating DNA replication in eukaryotic cells (18, 19). Our proteomics and GSEA analysis showed that MCMs involved in inhibition of cell proliferation through DNA Replication, Cell Cycle pathways, thereby we further verified these protein expressions on GBC-SD and NOZ cells after knocking down STK31 by Western blot. Additionally, the ITG family of proteins, also known as integrins, are a type of transmembrane receptor protein that is widely present on various cell surfaces and plays a central role in cancer cell invasion and metastasis (20). The result from proteomics also displayed ITG expressions decreased in shSTK31 group which might be responsible for reduced abilities of cell migration. Not surprisingly, as shown in Figure 4, *A* and *B* and Figures S3 and S4, shSTK31 significantly increased expression of MCM2, 3, 4, and 7 and reduced expression of CDC7, ITGB1, and ITGA3 relative to shNC. Due to the blockade of the G1 phase in cells after knocking down STK31, Cyclin D1/CDK4 expression, which are activated during early G1 phase, was also detected in our study. And the expressions of Cyclin D1 and CDK4 were significantly decreased after knocking down STK31 (Fig. 4, *A* and *B*). Given that the abnormal activation of MCMs was involved in the regulation of polyploidy formation (21, 22), and that we observed, following shRNA transduction, not only reduced cell proliferation but also evident morphological changes, including a marked increase in cell size (Fig. S5). Therefore, we further investigated the effect of polyploidization on the cellular response to knockdown of STK31. With 48 h of demecolcine treatment, the results of flow cytometry found that the percentages of wild type of GBC-SD cells with 4N and 8N DNA contents were 36.6% and 15.85%, respectively. And cells with 4N and 8N DNA contents were 46.54% and 15.45%, respectively, for shNC GBC-SD; 53.73 and 11.76%, respectively, for shSTK31-05; 57.17 and 11.38%, respectively, for shSTK31-06 (Fig. 4*C*). The above data revealed that after STK31 knockdown, the proportion of cells forming tetraploidy increased obviously.

**Figure 4:**
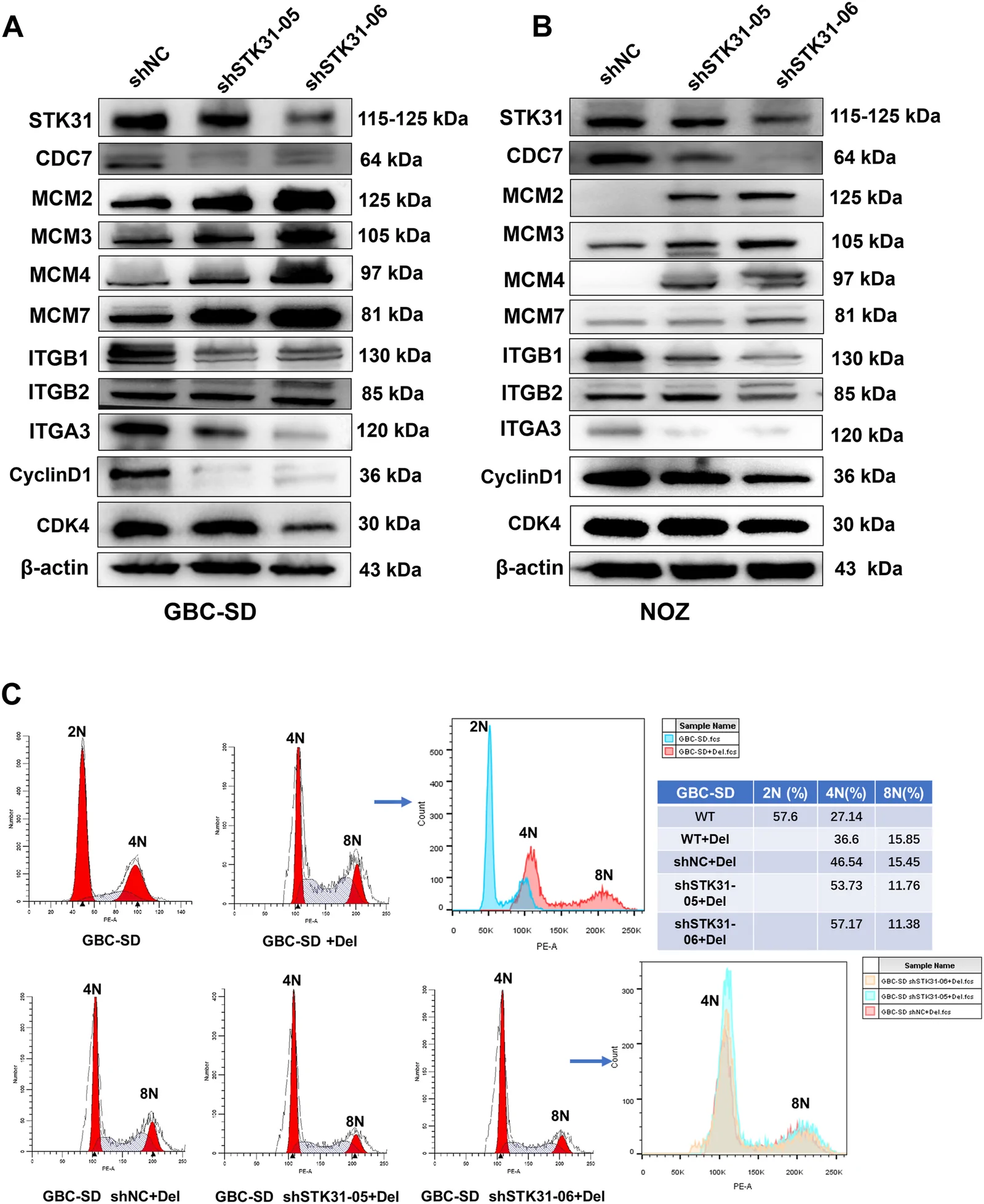
**Western blot validation of selected protein expressions, including MCMs, ITGs, and cell cycle related proteins CDC7, CyclinD1 and CDK4 in shSTK31 knock-down GBC-SD and NOZ cells.** Immunoblotting was performed three times using independently prepared whole cell lysates. *A* and *B*, representative immunoblots showing the effects of knock-down on GBC-SD and NOZ cellular expression of MCM2,3,4,7 and ITGB1, B2, A3, as well as CDC7, CyclinD1 and CDK4. β-actin expression was determined to confirm equal protein loading. *C*, knockdown of STK31 induces polyploidy. DNA content flow cytometric histograms of shNC, shSTK31-05, and shSTK31-06 GBC-SD with or without exposure to demecolcine (62.5 ng/ml) for 48 h.

### STK31 depletion facilitates the formation of polyploid cells through its direct interaction with c-myc and FOXP1

To explore the molecular mechanism underlying the regulation of STK31, we performed co-immunoprecipitation/massspectrometry (Co-IP/MS) to identify proteins that interacted with STK31 directly. Moreover, STK31 is a protein belonging to the serine/threonine kinase family, which regulates cellular function through phosphorylation (14). Therefore, we also analyzed the kinase activity of the recombinant human STK31 protein and identified phosphorylated proteins by proteomics after overexpression of STK31 in GBC-SD cells (Fig. S6, *A* and *B*). The results showed that STK31 possesses certain kinase activity (Fig. 5*A*). To test this hypothesis, the results from Co-IP/MS and phosphorylated proteomics for STK31 were subjected to intersection analysis, leading to the identification of 33 proteins (Fig. 5, *B* and *C*). Intriguingly, among these proteins, an interaction between STK31 and c-Myc, the transcription factor that regulates DNA replication through the promotion of cell cycle progression and proliferation, was found. In addition, FOXP1 had also been found to directly interact with STK31 by Co-IP/MS and phosphorylated proteomics (Fig. 5, *B* and *C*). These interactions were further confirmed by Co-IP/Western blotting (Fig. 5*D*). Additionally, we examined the cellular localization of STK31 and c-Myc using immunofluorescence, revealing colocalization of STK31 and c-Myc on the cell cytoplasm and partially in the nuclear (Fig. 5*E*). Subsequently, STK31 knockdown experiments revealed that STK31 depletion increased the expression of c-Myc and FOXP1 (Fig. 5*G*). Previous study showed that c-Myc was closely related to polyploid formation (22). And FOXP1 binds to the c-Myc promoter and represses its transcription, whereas under specific pathological conditions, c-Myc can upregulate FOXP1 expression *via* direct or indirect mechanisms (23, 24). Based on the experimental results of polyploid formation mentioned earlier, these observations indicate that depletion of STK31 may contribute to the polyploidization of GBC cells through enhancing c-Myc and FOXP1 expression. Notably, STRING protein interaction analysis also demonstrated a significant correlation between c-Myc expression and members of the ITG family (Fig. 3*E*). Further investigation demonstrated that STK31 regulated the phosphorylation of c-Myc at Thr244 and Ser62. Specifically, STK31 knockdown reduced c-Myc phosphorylation at Thr244 while increasing phosphorylation at Ser62, whereas STK31 overexpression exerted the opposite effects (Fig. 5, *F* and *G*). Previous studies have shown that c-Myc Ser62 phosphorylation stabilizes c-Myc and phosphorylation on Thr244 results in reduced c-Myc stability (25, 26). Therefore, we speculated that the changes in total c-Myc protein levels were caused by STK31-mediated alterations in its phosphorylation status. Based on the conserved domain prediction from the NCBI Conserved Domain Database (CDD) and Uniprot, residues 805 to 945 or 710 to 1019 encompass the predicted protein kinase catalytic domain of STK31. Ser854 is present instead of the conserved Asp which is expected to be an active site residue (Uniprot). Therefore, to further define the regulatory phosphorylation site and the essential kinase domain of STK31, we generated phosphomimetic (S854D), phosphodead (S854A), and kinase-domain truncation (ΔKD) mutants for functional characterization (Fig. S6*C*). Our results demonstrated that, compared to wild-type STK31, the ΔKD mutant exerted a strong inhibitory effect on c-Myc Thr244 phosphorylation, while the S854A mutant showed only a modest inhibition. In contrast, the S854D mutant exhibited a phosphorylation pattern comparable to that of the wild-type STK31 (Fig. 5*H*).

**Figure 5:**
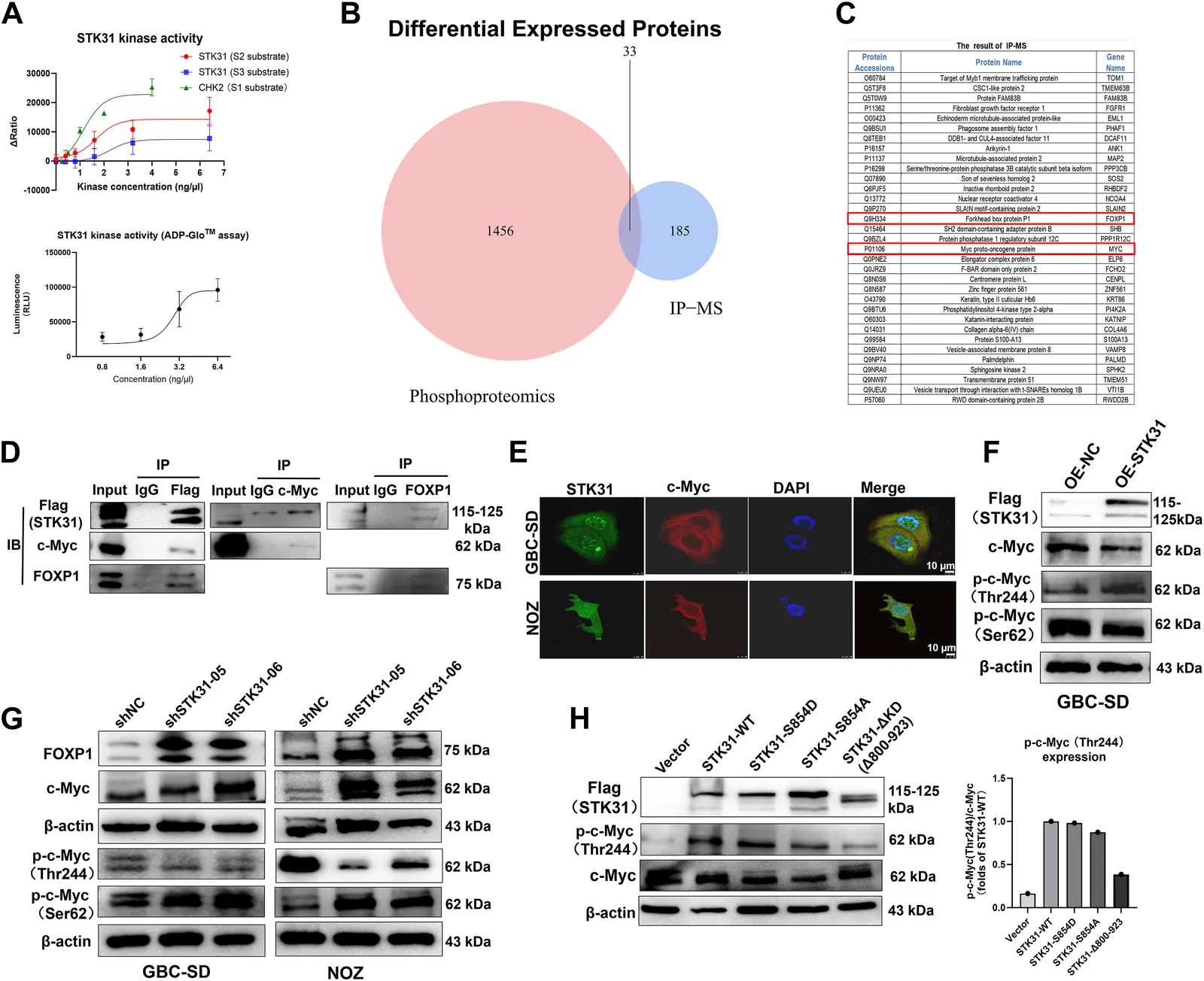
**STK31interact with c-Myc and FOXP1 to induce their expression**. *A*, kinase activity of STK31 was analyzed with HTRF KinEASE STK discovery kit (containing biotinylated STK substrates (S1, S2, and S3)) and P-Glo Kinase Assay. CHK2 was employed as a positive control. The experiment was independently repeated three times. *B*, Venn diagram comparing proteins that were significantly differential expressed (total 1489 proteins) in GBC-SD cells overexpressing STK31 compared to NC (FC>1.5; *p* < 0.05), proteins detected though Co-IP/MS in GBC-SD cells (total 218 proteins, excluding non-specific proteins identified in the IgG control group). *C*, 33 proteins identified from Co-IP/MS and phosphorylated proteomics were listed in this table. *D*, co-immunoprecipitation (Co-IP) of endogenous STK31 in GBC-SD cells using an anti-STK31 antibody, followed by IB analysis revealing the presence of c-Myc and FOXP1 in the STK31 Co-IP complex. Endogenous Co-IP/western blotting experiment performed in GBC-SD cells using c-Myc and FOXP1 antibodies. *E*, immunofluorescence used to observe the co-localization of STK31 and c-Myc in GBC-SD and NOZ cells. *F*, Western blot was used to detect the expression of c-Myc, p-c-Myc (Thr244), p-c-Myc (Ser62) after overexpressed of STK31. (*G*) Western blot was used to detect the expression of c-Myc, p-c-Myc (Thr244), p-c-Myc (Ser62) and FOXP1 after knockdown of STK31. β-actin expression was determined to confirm equal protein loading. The experiments were performed in three independent replicates. *H*, STK31 mutants differentially regulate c-Myc Thr244 phosphorylation. Immunoblots of cell lysates expressing the indicated STK31-Flag constructs. The ΔKD mutants, but not the phosphomimetic S854D, significantly impaired c-Myc Thr244 phosphorylation. The kinase-inactive S854A showed only a modest inhibition. β-actin, c-Myc and anti-Flag (STK31-Flag) blots are shown as loading and expression controls, respectively.

### STK31 depletion suppressed GBC tumor growth in xenograft models

Since STK31 knockdown showed inhibiting effects on GBC in *in vitro* studies, further animal studies were carried out. Tumor xenografts developed successfully in nude mice after subcutaneous injection with GBC-SD and NOZ cells (Fig. 6*A*). After inoculation for 24 or 25 days, the tumor sizes of shSTK31 group mice were, in general, smaller than those of the shNC group (Fig. 6, *C*–*F*). *In vivo* study also showed that tumor weight was significantly decreased in tumor-bearing mice inoculated with knocking down STK31 cells (Fig. 6, *G* and *H*). Notably, we observed in NOZ transplantation tumor model, tumors formed in only three of the five recipient mice (Fig. 6*B*). These results indicated that STK31 depletion restrained GBC-SD and NOZ tumor growth as well as tumorigenesis. The tumorigenicity was primarily affected by the significant impairment of cell proliferation after knocking down STK31, which was consistent with the *in vitro* experiments.

**Figure 6:**
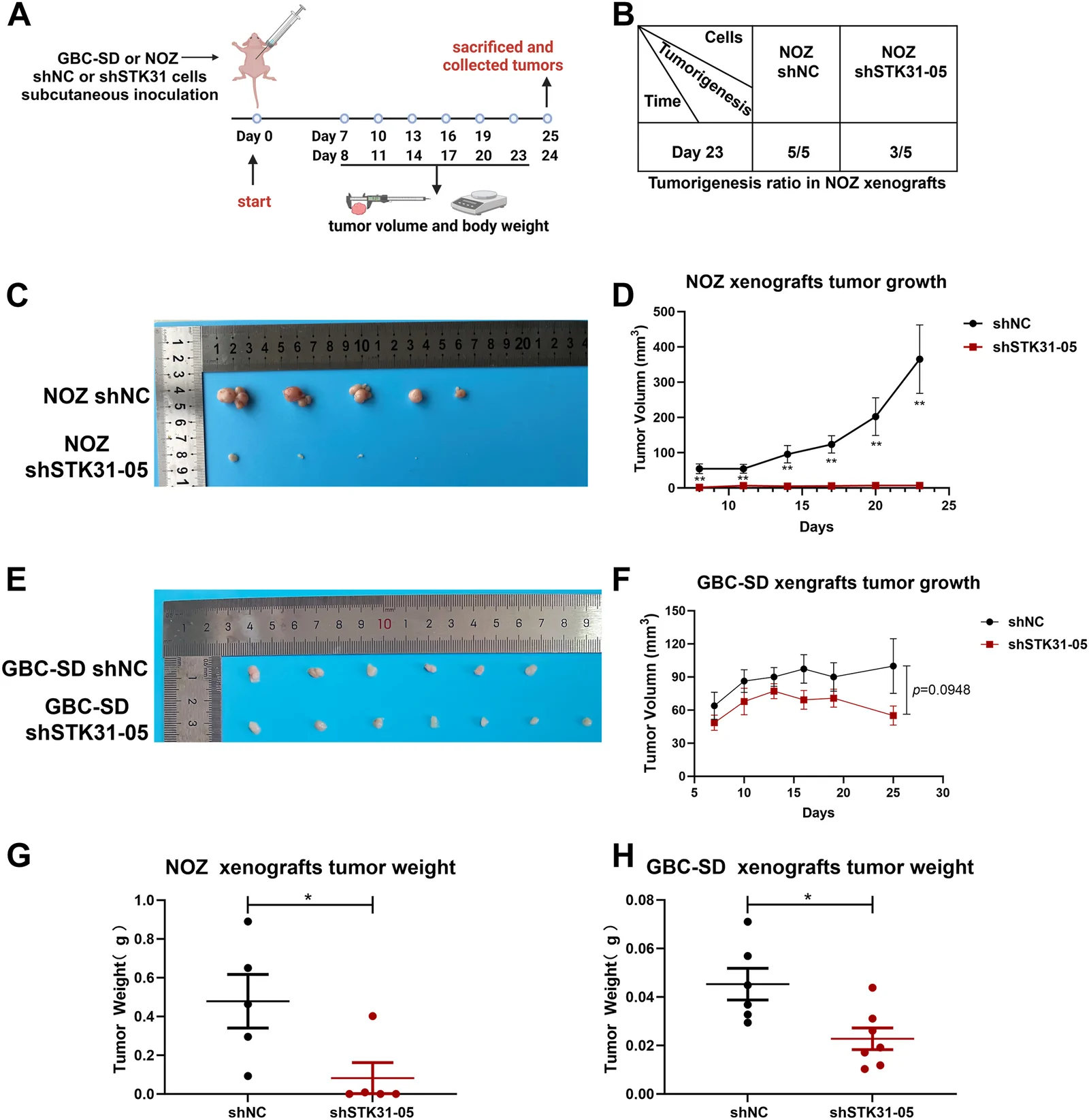
**STK31 depletion suppressed NOZ and GBC-SD xenografts tumor growth**. *A*, experiment timeline of the *in vivo* model (Graph was Created in https://BioRender.com). *B*, the tumorigenesis of NOZ xenografts after knock-down STK31. *C* and *E*, the representative image of the NOZ and GBC-SD xenografts tumors dissected from the mice. *D* and *F*, effects of knocking down STK31 on growth of xenograft tumors. *G* and *H*, each tumor was weighed and recorded. Data are shown as individual values and were compared with unpaired *t* test, n = 5 to 7, ∗*p* < 0.05, ∗∗*p* < 0.01.

### ATO interacts with STK31 and inhibits GBC progression *in vitro* and *in vivo*

Our findings establish STK31 as a potential therapeutic target for GBC, however, specific inhibitors of STK31 are currently unavailable. Subsequently, we constructed a three-dimensional structural model of the complex using AutoDock Vina software based on the AlphaFold Protein Structure Database of UniProt and ChEMBL database. Among 1008 small molecular compounds, the binding free energy values (docking score) of 209 compounds with STK31 were below −8.0 kcal/mol (Fig. 7*A*). Especially, the binding free energy values of Atovaqueone (ATO) with STK31 were −9.6 (optimized to −9.9kal/mol), which draws our attention (Fig. 7, *B* and *C*). The molecular docking prediction results also revealed that ATO can bind to the kinase domain of STK31 (Fig. 7*C*). ATO is a kind of hydroxynaphthoquinone drug, which was initially developed to treat and prevent infectious diseases caused by protozoa. Recent studies have found that ATO could inhibit different tumors including colorectal cancer, breast cancer, ovarian cancer, gastric cancer and thyroid cancer *etc.* through inhibiting mitochondrial respiration, Wnt and NFκB signaling pathways (11, 13, 27, 28). Hence, we next validated whether ATO targets to STK31 by CETSA in GBC-SD cell lysates. The result revealed that ATO indeed increased thermal stability of STK31 when cell lysates were heated from 37 °C to 72 °C, indicating a drug–protein interaction between ATO and STK31 (Fig. 7*D*). The inhibitory effect of ATO on the protein level of STK31 was confirmed by Western blot using different concentrations. ATO could significantly inhibit the protein expression of STK31 at doses higher than 25 μM (Fig. 7*E*). Additionally, ATO also suppressed the expression of ITGA3 and ITGB1 (Fig. 7*E*). Moreover, the *in vitro* and *in vivo* antitumor activities of ATO on GBC was further evaluated. As shown in Figure 7*F*, CCK-8 assay showed that ATO suppressed cell proliferation dose-dependently and time-dependently in two GBC cell lines, GBC-SD and NOZ. Overexpression of STK31 significantly attenuated the ATO-induced inhibition of GBC-SD cell viability at both 2 and 3 days of treatment (Fig. 7*G*). Meanwhile, the kinase activity of STK31 was significantly inhibited by ATO treatment (Fig. 7*H*). Concurrently, we treated both STK31 ΔKD mutant and wild-type STK31 cells with ATO (25 μM and 50 μM) for 2 days and found that the ΔKD mutant significantly promoted cell proliferation compared to the wild-type group (Fig. 7*I*). Moreover, CETSA indicated a drug–protein interaction between ATO and STK31-WT, whereas this interaction was no longer present in the STK31 ΔKD mutant (Fig. S7, *A* and *B*). Further experiments showed impaired clone formation abilities after treatment with ATO (Fig. 8, *A*–*C*). In the presence of ATO from 2.5, 25 and 50 μM, cell migration of GBC-SD cells reduced by 34.30 ± 11.46%, 64.20 ± 20.92%, 67.03 ± 9.30%, respectively; cell migration of NOZ cells declined by 29.35 ± 24.83%, 56.13 ± 34.66%, 75.36 ± 21.97%, respectively (Fig. 8, *D*–*F*). *In vivo* results demonstrated that oral administration of ATO had significant inhibitory effects on tumor volume and weight compared to the vehicle control (Fig. 8, *G*–*J*). Meanwhile, the body weight of the mice was not affected by ATO, indicating no significant toxicity (Fig. S7*C*).

**Figure 7:**
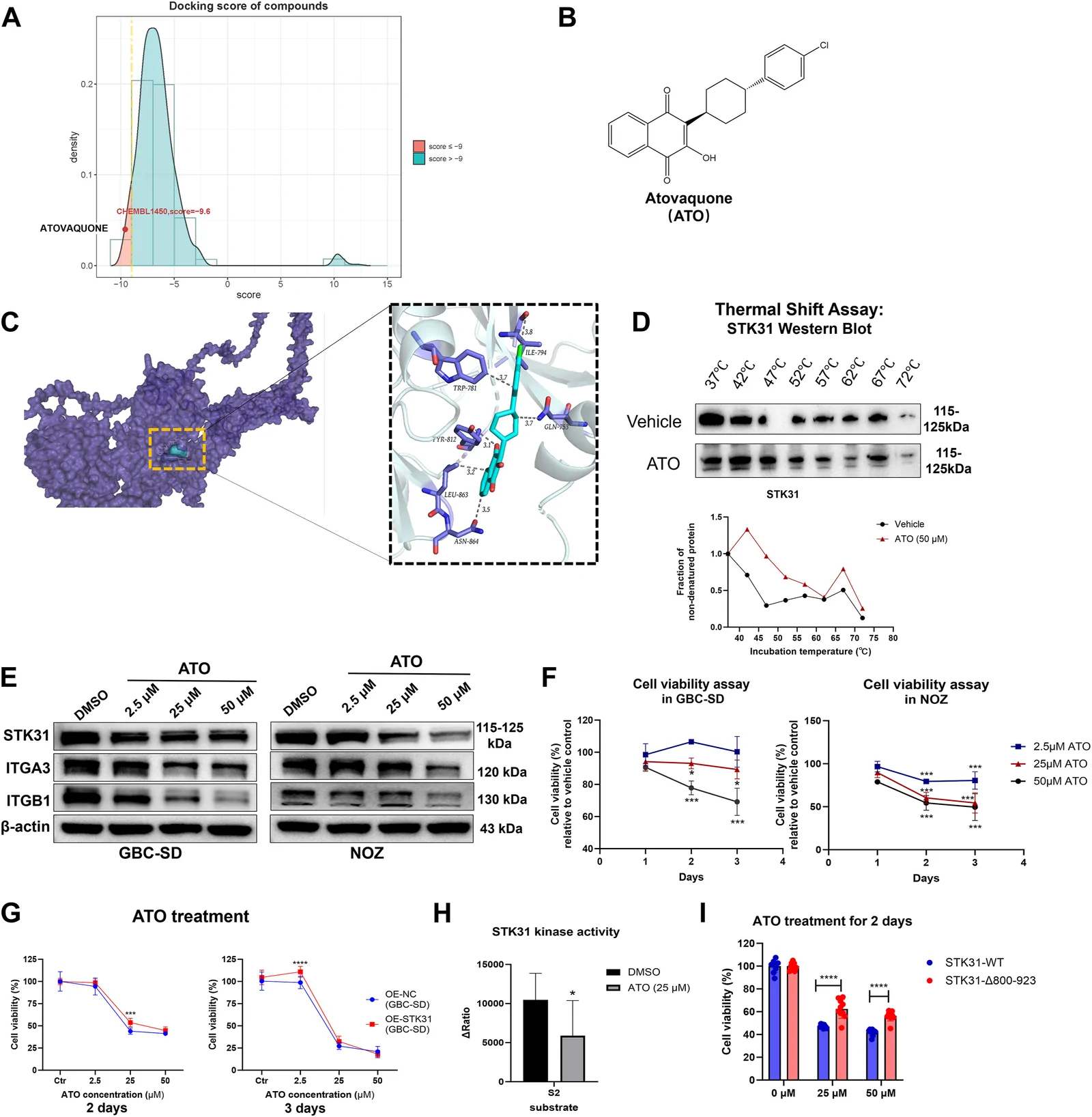
**Identification of potential inhibitors of STK31**. *A*, using the structure of STK31 in the AlphaFold Protein Structure Database of UniProt in combination with small molecule compounds from ChEMBL database, the total 1008 compounds were screened, and among them 209 compounds with a docking score less than −8.0 were obtained. The compound atorvaquone (CHEMBL1450) with a docking score of −9.6 (optimized to −9.9kal/mol) was indicated in distribution map, which was plotted by the ggplot2 package in R language. *B*, chemical structure of Atorvaquone (ATO). *C*, three-dimensional structure of the molecular docking of ATO and STK31. The binding of ATO and STK31was simulated by using Pymol software. The *red* color, the *purple* color and the bright *blue* color represent oxygen atoms, carbon atoms in amino acids, and the carbon structure of ATO, respectively. The dashed line represents the distance between corresponding atoms, measured in Å. *D*, cellular thermal shift assay for validation of ATO interacted with STK31. GBC-SD cell lysates were incubated with 50 μM ATO and heated at different temperatures for 10 min, then subjected to immunoblotting. Thermal shift curves of ATO target with increased thermal stability by STK31 immunoblotting in GBC-SD cell lysates. Graph indicates STK31 band intensities quantified using ImageJ. *Red* curves represent ATO treatment and *black* curves represent vehicle treatment. *E*, Western blot was used to analysis the expression of STK31, ITGA3, and ITGB1 after ATO treatment in GBC-SD and NOZ cells. β-actin expression was determined to confirm equal protein loading. *F*, ATO inhibited cell viability. ATO on the cell viability of GBC cell lines (GBC-SD and NOZ). Different concentrations of ATO treatment were initiated 24 h after seeding the cells in 96-well plates. Cell viability was assessed by the CCK-8 assay after 24, 48 and 72 h of ATO treatment. Results were expressed as percentages of CCK-8 absorbance with respect to the untreated vehicle control wells (mean ± SD of four independent experiments with five wells each). Differences between ATO treated groups and vehicle control group were determined by one-way ANOVA followed by *post hoc* Dunnett’s test, ∗∗∗*p* < 0.001. *G*, GBC-SD cells overexpressing STK31 exhibited reduced sensitivity to ATO at 2 and 3 days post-treatment. Data are representative of three independent experiments. *H*, ATO inhibited kinase activity of STK31 (mean ± SD of three independent experiments). Differences between ATO treated groups and vehicle control group were determined by unpaired *t* test, ∗*p* < 0.05. *I*, the STK31 ΔKD mutant attenuated the inhibitory effect of ATO on GBC-SD cells. CCK8 assay showed that after 2 days ATO treatment, the STK31 ΔKD mutant exhibited a significant increase in cell proliferation relative to wild-type STK31 cells. Differences between the STK31 ΔKD mutant and wild-type group were determined by unpaired *t* test, ∗∗∗∗*p* < 0.0001.

**Figure 8:**
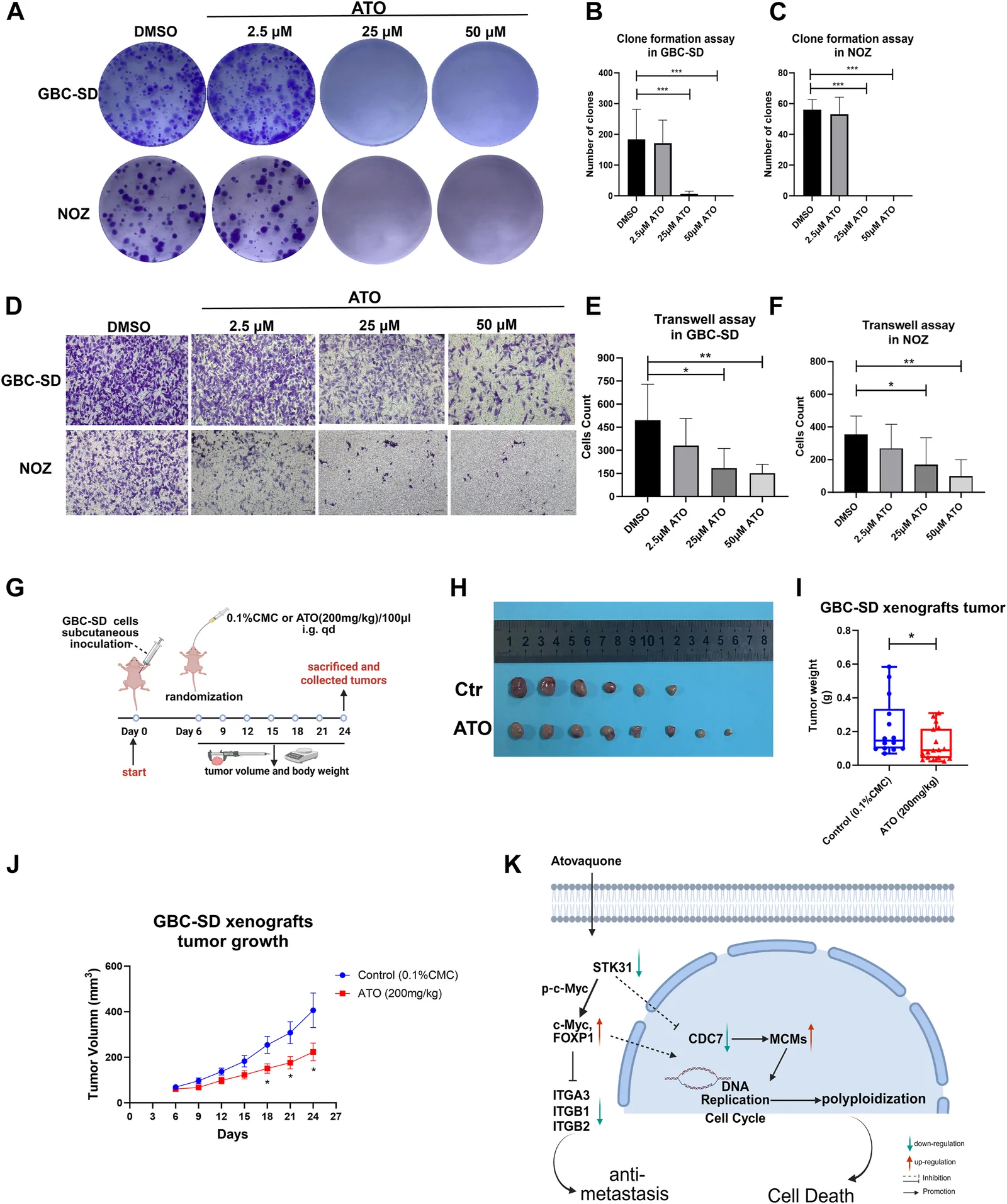
**ATO inhibited clone formation and migration of GBC-SD and NOZ cells, and the anti-tumor efficacy of ATO in GBC-SD subcutaneous xenograft bearing nude mice**. *A*, the representative photos of clone formation assay. *B* and *C*, bar graphs showing the clones counted in four independent experiments. *D–F*, effects of ATO on migration of GBC-SD and NOZ cells in Boyden chambers. *D*, representative photomicrographs showing the stained cells on the lower side of the membrane. The cells in the *upper* chambers were treated with different doses of ATO. After 12 to 16 h incubation, those cells migrated to the lower chambers were stained and the numbers were counted. *E* and *F*, quantification of migration of GBC-SD and NOZ cells. Results are expressed as the mean percentage of control (mean + SD of three independent experiments with two wells each). Differences between ATO treated groups and vehicle control group were determined by one-way ANOVA followed by *post hoc* Dunnett’s test, ∗*p* < 0.05, ∗∗*p* < 0.01, ∗∗∗*p* < 0.001. *G*, scheme depicting the experimental setup of the *in vivo* animal model Graph was Created in https://BioRender.com. *H*, photograph showing the representative tumors in ATO treatment group. *I*, effect of ATO treatment on tumor weight after 19 days of treatment. *J*, effect of ATO on growth of xenograft tumors (n = 14–17). Data are shown as individual values and were compared with unpaired *t* test, ∗*p* < 0.05. *K*, schematic representation of the inhibitory mechanisms of STK31 in GBC (Graph was Created in https://BioRender.com).

## Discussion

STK31, a newly identified CTA, is normally expressed only in the testes and is absent in other healthy tissues (5). However, it is aberrantly overexpressed in various types of tumor tissues, particularly in gastrointestinal cancers, and may serve as a driver gene for gastrointestinal tumors (29, 30). Therefore, it is considered a potential ideal target for cancer therapy and diagnostic biomarker (31). Despite its low incidence, GBC is characterized by high malignancy and a typically insidious onset. The lack of specific therapeutic targets and early diagnostic biomarkers is a major contributor to GBC’s poor prognosis (3). Our study provided the first evidence that STK31 is overexpressed in GBC and drives tumorigenesis and progression.

Although TCGA data did not show a significant negative correlation between STK31 overexpression and prognosis in GBC—likely due to its low incidence and limited sample size—expanded analyses across pan-cancer and gastrointestinal malignancies revealed a strong association between elevated STK31 expression and unfavorable clinical outcomes.

While STK31 was previously implicated in DNA replication, its precise mechanistic role was undefined (6, 9). Our proteomics data unexpectedly linked STK31 to the MCM complex, yet IP-MS and Co-IP experiments showed this regulation is indirect—a finding that reshapes understanding of its replicative functions. MCMs are loaded onto DNA during the G1 phase to form pre-replication complexes, which then promote DNA unwinding and initiate replication during the S phase (16, 18, 19). STK31 knockdown markedly inhibited GBC-SD cell proliferation and led to a pronounced increase in cell size (Fig. S5). Coupled with the elevated expression of MCM family proteins, these results indicate that STK31-depleted cells initiated DNA replication but failed to complete cytokinesis, thereby potentially promoting the formation of polyploid cells. Our flow cytometry analysis conclusively demonstrated the formation of polyploid cells following STK31 knockdown. In certain contexts, inducing polyploidy in tumor cells may represent an anticancer strategy, as polyploid cells are more prone to stress responses, ultimately undergoing senescence or apoptosis (32). Certain chemotherapeutic agents (*e.g.*, paclitaxel and vinblastine) disrupt microtubule function, causing aberrant mitosis and polyploidization, ultimately triggering apoptotic cell death (33, 34). Collectively, our findings demonstrate that STK31 depletion recapitulates the tumor-suppressive effects observed in other polyploidization-based therapies, establishing its therapeutic potential for GBC.

CDC7 is frequently overexpressed in tumor cells, where it plays a critical role in initiating DNA replication and maintaining orderly cell cycle progression (35). Its inhibition or dysregulation can impair replication processes, leading to replication stress or the development of aneuploid cells (36). CDC7 is a serine/threonine protein kinase that initiates DNA replication by phosphorylating the MCM complex, thereby activating the DNA helicase and licensing replication origins (16, 17). Our study demonstrates that STK31 knockdown reduces CDC7 expression. Building upon our previous findings that STK31 depletion induces G1 cell cycle arrest, we propose that STK31 may regulate cell cycle progression and polyploidization by modulating CDC7-mediated phosphorylation of the MCM complex. During G1 cell cycle arrest, both the expression and the kinase activity of Cyclin D1 and CDK4 are significantly downregulated (37, 38). Therefore, our data revealed that STK31 knockdown induced G1/S phase arrest in a manner dependent on the downregulation of CDK4 and Cyclin D1 expression.

The c-Myc transcription factor critically regulates DNA replication by directly activating genes involved in cell cycle progression (including cyclins, CDKs) and replication licensing (MCM2-7), thereby coordinating proliferation with genome duplication (39). Although c-Myc is conventionally classified as an oncogene, overexpression of c-Myc has been shown to induce polyploidization by promoting DNA replication in the absence of proper mitotic progression, often leading to mitotic slippage or cytokinesis failure (22). Mitotic disruption-induced polyploidy eventually causes cell proliferation blockage and death. This explains why, despite elevated c-Myc expression, STK31-depleted GBC cells still showed proliferation arrest and eventual cell death. Moreover, FOXP1 may dysregulate key factors in cell cycle phase (40). Our experimental data suggest that STK31 likely interacts directly with both c-Myc and FOXP1 in a phosphorylation-dependent manner. Many studies have shown that c-Myc Ser62 phosphorylation stabilizes c-Myc and phosphorylation on Thr244 results in reduced c-Myc stability (25, 26). To this end, we evaluated the phosphorylation status of c-Myc. Our results demonstrated that STK31 regulates phosphorylation at Ser62 and Thr244, consequently altering total c-Myc protein abundance. However, the absence of commercially available phospho-specific antibodies against FOXP1 currently limits our ability to fully elucidate its mechanism. UniProtKB/Swiss-Prot annotates that Ser854 replaces the conserved catalytic Asp residue expected in the active site of conventional protein kinases, suggesting that STK31 possesses only limited kinase activity. In our assays, we confirmed that STK31 does exhibit detectable kinase activity; however, when compared to the positive control CHK2, its enzymatic activity appeared relatively modest. Thus STK31 exerts a direct positive regulatory effect on c-Myc phosphorylation at Thr244. Notably, STK31 expression also correlated with altered phosphorylation at Ser69, raising the possibility of an indirect regulatory mechanism. This is particularly plausible given our recent finding that STK31 directly modulates GSK3β phosphorylation (41), which in turn has been implicated in the regulation of c-Myc Ser69 phosphorylation. Additionally, Integrin-mediated cell-ECM adhesion promotes tumor cell migration and invasion (20). Our study also found that knocking down STK31 significantly suppressed ITGA3 and ITGB1, which may be the primary reason for STK31's effect on cell migration.

Molecular docking is a computational method used to predict the binding modes and interaction sites between molecules, such as drugs or ligands, and target proteins (42). Therefore, after elucidating the role of STK31 in GBC, we performed molecular docking-based screening of 1008 small molecules from the ChEMBL database. Among the screened compounds, ATO exhibited a notable binding free energy of −9.9 kcal/mol with STK31, attracting our attention. ATO, a synthetic hydroxy-1,4-naphthoquinone derivative, is widely used as an antimicrobial agent. ATO’s chemical structure resembles that of coenzyme Q, a structural mimicry enabling it to target mitochondrial complex III and disrupt electron transport chain function (13). Due to its mechanism of disrupting mitochondrial function, ATO has demonstrated efficacy not only in antiparasitic therapy but also exhibits potential in cancer treatment. Recent studies suggested that ATO may exert anticancer effects by interfering with energy metabolism, cell proliferation, and anti-apoptotic pathways in cancer cell mitochondria (11, 12, 28). However, the molecular mechanisms underlying its antitumor effects have yet to be fully elucidated. We demonstrated for the first time that ATO directly binds to STK31 and downregulates its kinase activity and expression, which may represent one of the key mechanisms underlying its anti-GBC effects. Molecular docking, CETSA, and enzyme activity assays all confirmed that ATO targets STK31; however, how ATO also inhibits STK31 protein expression remains unknown. We speculate that additional mechanisms, such as transcriptional repression or protein degradation, may be involved, and this warrants further investigation. Based on our results, ATO should be considered as a promising candidate for further development into an efficacious agent for GBC treatment.

## Conclusion

Taken together, our study provides the first evidence that STK31 promotes GBC progression by binding to c-Myc/FOXP1 and regulates the phosphorylation state of c-Myc *via* its kinase activity, thereby modulating DNA replication and its associated regulatory proteins (Fig. 8*K*). Inhibition of STK31 induces polyploidization in cancer cells, highlighting its potential as a therapeutic target. Notably, ATO was identified as a potential STK31 inhibitor, supporting its further evaluation as a therapeutic agent against GBC.

## Experimental procedures

### Materials

Human gallbladder cancer cells GBC-SD and NOZ were purchased from the Cell Bank of Type Culture Collection of Chinese Academy of Sciences, which were authenticated by Short Tandem Repeat profiling before use in the experiments. Antibody against STK31 for Western blot was obtained from Biobyt. A lentiviral short hairpin RNA (shRNA) targeting STK31 (puromycin-resistant) and STK31 overexpression lentiviral vector (Flag-tagged) were purchased from Genechem. The interference sequence targeting human STK31 complementary DNA is shSTK31 to 04: CCGGCCTCTGTAGCTTGATATGTTACTCGAGTAACATATCAAGCTACAGAGGTTTTTG; shSTK31 to 05: CCGGGCTATCCATTAAGAAGACATTCTCGAGAATGTCTTCTTAATGGATAGCTTTTTG; shSTK31 to 06:CCGGCAAGCCAACATGCCTTTAAATCTCGAGATTTAAAGGCATGTTGGCTTGTTTTTG and a scramble shRNA was included as negative control (shNC): 5′-TTCTCCGAACGTGTCACGT-3′. An STK31 overexpression lentiviral vector (puromycin-resistant) was built using the sequence: AGGTCGACTCTAGAGGATCCCGCCACCATGTGGGTCCAGGGTCACTCTTCTAGAG. CCK-8 Kit, primary antibodies against MCM2, 3, 4, 7, ITGB1, ITGB2, ITGA3, c-Myc, CDK4, Cyclin D1, CK19, Flag and β-actin were purchased from Proteintech, Rosemont, IL 60018, USA. Antibody against p-c-Myc (Ser62) was purchased from PTMBIO. The antibodies against FOXP1, CDC7, p-c-Myc (Thr244) and Protein A (HRP Conjugate) were obtained from Cell Signaling Technologies. Anti-STK31 polyclonal antibody for immunohistochemistry and immunofluorescence staining was from Invitrogen. Atovaquone (ATO) was purchased from Medchemexpress and dissolved in DMSO or 0.1% CMC (Solarbio) before administration.

### Patients and clinical sample

The GBC tissue microarray analyses (TMA) was purchased from Shanghai Outdo Biotechnology company. The Five paired fresh-frozen GBC tissues and normal paracarcinoma tissues from patients who underwent radical surgery without preoperative chemotherapy or radiotherapy were collected for TMA. Paraffin-embedded tissues from 72 cases of GBC and 108 cases of chronic cholecystitis, were obtained and cut into 4-mm sections for further analysis (43). Six patients with gallstones received cholecystectomy surgery, and the gallbladder mucosa was collected for further primary culture. 15 patients with GBC also received cholecystectomy surgery, and the GBC tissues and normal paracarcinoma tissues were collected for real-time PCR. Paraffin-embedded tissues from 6 GBC cases and freshly resected tissues from one GBC case were also used for immunohistochemistry and Western blot analysis, respectively. Informed consents were obtained from all the patients, and samples collection was complied with the approved guidelines of Ethics Committee of Yanan Hospital affiliated to Kunming Medical University (2022–026–01). The impact of gene *STK31* on overall survival in pan-cancer and digestive system tumors, as well as its differential expression between tumor and adjacent normal tissues, was also analyzed using data from the TCGA database and the GEPIA website.

### RNA extraction and real-time PCR

Real-time PCR were performed to examine the expression of the *STK31* gene in GBC tissues and normal paracarcinoma tissues. The total RNA was extracted from the tissues by using TRIzol reagent (Life Technologies Inc) according to the manufacturer’s instruction after tissue grinding in liquid nitrogen. Then the RNA was extracted and purified by NucleoSpin RNA clean-up kit (Macherey-Nagel). cDNA was synthesized by GoScript Reverse Transcription Mix, Oligo(dT) based on the manufacturer’s instruction. The concentration of RNA and cDNA were determined by a spectrophotometer (DeNovix). Real-time PCR was conducted by CFX96 Real-Time System and SsoFast EvaGreen Supermix (Bio-Rad, CA, USA), and the specific gene mRNA levels were normalized to GAPDH in accordance with ΔΔCq method and expressed as a fold change compared to the normal group. Melting curve analysis was utilized to confirm amplification specificity of the PCR products. The primers used were as follows:STK31 forward (F), 5′-TGAACTCTGGTGGTCTCCTTAC-3′, and reverse (R), 5′-CTTGGCTTCTGTGTCAACATCC-3′; GAPDH F, 5′-GGAGCGAGATCCCTCCAAAAT-3′, R, 5′-GGCTGTTGTCATACTTCTCATGG-3′.

### Microarray expression profiling and bioinformatic analysis

Long non-coding RNA (lncRNA) and mRNA TMA profiling of GBC and paracarcinoma samples was performed according to our previous study (43). Briefly, it was conducted by Beijing Capitalbio Technology company on a SurePrint G3 Human Gene Expression 8 × 60Kv2 Microarray (Agilent Technologies). Raw data were acquired and input into GeneSpring GX software (Agilent Technologies), and normalization was processed using the percentile shift method. Volcano plot produced by R language and ggplot2 was used to visualize differentially expressed genes (DEGs, ∣log2(fold change) ∣ ≥ 1, *p* value < 0.05).

### Immunohistochemistry (IHC)

Paraffin-embedded sections from patients were processed according to our previous study (43). The sections were subjected to immunostaining to determine the expression of STK31 in the tumors. The antibody against STK31 was applied to sections at 1:1000 dilution in Antibody Diluent (DAKO), which were incubated overnight at 4 °C. The secondary antibody (Biotinylated MultiLink; DAKO) was incubated by an Autostainer Link 48 machine for 30 min. Then the sections were applied with horseradish peroxidase enzyme complex to complete the staining. The slides were subsequently counterstained in hematoxylin for 3 min and finally mounted onto microscopic rapid-mounting media under glass coverslip.

### Cell culture and lentivirus infection

Normal epithelial cells in gallbladder mucosa were primary cultured according to previous study (44). Briefly, tissue from the mucosal epithelial layer was cut into approximately 1-mm^3^ pieces and laid on a 6-well plate. The tissue was cultured with Dulbecco’s Modified Eagle Medium (DMEM)/F12 supplemented with 20% fetal bovine serum (FBS) after keeping them in a 37 °C incubator for 1 h. The culture medium was replaced every 2 days. When the cell density reached approximately 70%, the epithelial tissue was carefully removed using sterile forceps. The cells were cultured continuously for another 1 to 2 days until the cell density reached around 80%, then proceed with the first passage. The identification of primary cultured epithelial cells was carried out through detection of CK19 by immunofluorescence staining. GBC-SD and NOZ cells were cultured in RPMI 1640 and (DMEM)/F12 (Gibco, Thermo Fisher Scientific (SuZhou) Instruments) respectively, and supplemented with 10% FBS, and 100 U/ml penicillin and 100 μg/ml streptomycin (Vivacell). The cells were incubated at 37 °C in a 5% CO_2_, 95% humidified atmosphere. STK31 was knock-downed by specific short hairpin RNA (shRNA) and the stable GBC-SD and NOZ cell clones were selected by puromycin. STK31 overexpression was also conducted by lentiviral vector. Lentivirus infection was performed according to the manufacturer’s instructions. Subsequently, cells were co-cultured with 2 μg/ml puromycin for 7 days to screen out cells not expressing vectors.

Lentiviral vectors encoding human wild-type STK31 (NM_031414.5) and its mutants were synthesized and packaged by GeneChem Co., Ltd. The constructs included wild-type STK31 (3 × FLAG-STK31), the phospho-deficient mutant (STK31-S854A), the phospho-mimetic mutant (STK31-S854D), and the C-terminal deletion mutant (STK31-Δ800–923) (45). All overexpression constructs were generated using the GV513 lentiviral vector (Ubc-MCS-CBh-gcGFP-IRES-puromycin) with an N-terminal 3×FLAG tag. An empty vector control was constructed using the GV492 lentiviral vector (Ubc-MCS-3FLAG-CBh-gcGFP-IRES-puromycin) and served as a negative control. Stable overexpression of STK31 wild-type, mutants, and empty vector control in GBC-SD cells was confirmed by Western blot.

### Cell viability and colony formation assays

After lentivirus transduction, cells were trypsinized, resuspended, and seeded in 96-well plates (at 1 × 10^3^ cells/well). Cell viabilities were quantified for 4 days at the indicated time points by using CCK-8 assay according to the manufacturer’s instruction. Optical density (OD) at 450 nm absorbance was detected by a microplate spectrophotometer (Varioskan Lux, Thermo Scientific). For cell clonogenic capacity assays, the cells with stable knockdown of STK31(at 1 × 10^3^ cells/well) were seeded in 6-well plates in duplicate conditions. After 10 or 12 days of culture cells were fixed in methanol, stained with 1% crystal violet solution, air-dried, and the colonies were taken photos and counted.

In another set of experiment, cells were seeded at 1 × 10^3^ cells/well in 96-well plates for 24 h. Then ATO, dissolved in DMSO to produce a stock solution in advance, were diluted with culture medium to various concentrations and added to the cells. After 24, 48 and 72 h treatment, CCK-8 was added to each well for the detection of cell viability. Cell viability was expressed relative to vehicle control data. The cell clonogenic capacity after ATO treatment was also evaluated. NOZ at 5 × 10^2^ cells/well and GBC-SD at 1 × 10^3^ cells/well were seeded into 6-well plates, then different concentration of ATO was added into the cells for 10 or 12 days, respectively. The clone formation was detected through the similar preceding procedures.

### Cell migration assay

The migration abilities of GBC-SD and NOZ after knockdown of STK31 or treatment with ATO were evaluated by the Boyden chamber assay as described with our previous study (46). Briefly, cell suspension (shSTK31–05, shSTK31–06 and shNC, 5 × 10^4^ cells in 100 μl medium) was added into each transwell filter chamber with 8 μm pore size. Seven hundred microliters of culture medium with 10% v/v FBS, serving as chemoattractant medium, were added to the lower chamber. After incubation for 16 to 18 h at 37 °C, the cells on the bottom surface of the filter membrane were fixed with methanol and stained with 0.1% crystal violet dye solution. Stained filters were photographed under a light microscope (Primo Vert, Carl Zeiss Microscopy GmbH). The stained cells were counted manually by Image J software.

To assess the inhibitory effects of ATO on cell migration, another set of experiment was carried out by adding ATO (2.5, 25 or 50 μM) into the upper chambers when the cells were simultaneously seeded. After 12 to 16 h treatment, the migrated cells were fixed, stained and analyzed by the method mentioned above.

### Cell cycle and cell polyploid analysis by flow cytometry

Cell cycle percentages were evaluated with Cell Cycle and Apoptosis Analysis Kits (Beyotime). Cells were cultured in 6-well plates and collected after 7 days of shRNA transduction. Cells were trypsinized and washed with ice-cold PBS, and then permeabilized with ice-cold 70% v/v ethanol at 4 °C overnight. The permeabilized cells were resuspended in PBS and stained with propidium iodide solution containing RNase A for 30 min at 37 °C in the dark. After staining, cells were analyzed on a flow cytometer (FACScalibur; Becton Dickinson). Data were collected and calculated using Modfit software (http://www.vsh.com/products/mflt/index.asp).

To assess cancer cell polyploidization after knockdown of STK31, cells with or without demecolcine (62.5 ng/ml) were incubated for 48 h. Then the DNA content of shNC cells, shSTK31 cells, wild-type cells was also analyzed through flow cytometry after PI staining as described in the previous steps. In the analysis of demecolcine treated cells, where formation of polyploid population in the presence of demecolcine in GBC-SD cells, untreated control samples were analyzed in parallel as references to accurately distinguish ploidy levels. Data was analyzed and generated using Modfit software, and the images were plotted by FlowJo X program.

### Proteomics analysis

STK31-knockdown GBC-SD cells were subjected to 4D label-free quantitative proteomics for global protein expression analysis. Briefly, the peptide of each sample was desalted on C18 Cartridges, then concentrated by vacuum centrifugation and reconstituted in 40 μl of 0.1% (v/v) formic acid. The peptide content was estimated by UV light spectral density at 280 nm using an extinctions coefficient of 1.1 of 0.1% (g/l) solution that was calculated on the basis of the frequency of tryptophan and tyrosine in vertebrate proteins. 2 μg peptide was loaded onto the C18-reversed phase analytical column (Thermo Fisher Scientific, Acclaim PepMap RSLC 50um X 15 cm, nano viper, P/N164943) in buffer A (0.1% formic acid) and separated with a linear gradient of buffer B (80% acetonitrile and 0.1% Formic acid) at a flow rate of 300 nl/min. LC-MS/MS analysis was performed on a Orbitrap Exploris 480 mass spectrometer (Thermo Fisher Scientific) that was coupled to Easy nLC (Thermo Fisher Scientific).

Gene set enrichment analysis (GSEA) was performed to identify potential biological mechanisms associated with STK31 knockdown in GBC-SD. The proteomics expression profiling data visualizations, including correlation clustering, differenetial protein expression, and gene set enrichment analysis, were implemented using ggplot2 (3.5.1), dplyr (1.1.4), pheatmap (1.0.12), clusterProfiler (4.16.0) and fgsea (1.34.0) packages in R language (4.4.2). Statistical significance was prospectively defined as *p* < 0.05. The STRING database (http://string-db.org/cgi/input.pl) was employed for further understanding the protein–protein interaction (PPI) information involved in the DNA replication, Cell Cycle and ECM Receptor Interaction.

PRM-based targeted proteomics (LC-MS/MS) validated the altered expression of key proteins involved in DNA replication, cell cycle regulation, and ECM receptor interaction pathways. The samples were subjected to chromatographic separation followed by mass spectrometric analysis using a Q Exactive HFX mass spectrometer. The analysis was performed over a 60-min duration in positive ion mode, with precursor ions scanned over the m/z range of 350 to 1500.

### Western blot

GBC-SD and NOZ cells were harvested from dishes by gently scraping and were lysed with radioimmunoprecipitation assay buffer (RIPA, Beyotime) containing a protease inhibitor cocktail (Roche Molecular Biochemicals, Switzerland). The protein concentrations were determined by a bicinchoninic acid (BCA) assay (Beyotime). Protein aliquots of 30 μg were electrophoresed on 10% SDS-PAGE gels at 120 V. The separated proteins were transferred electrophoretically for 2 h at 200 mA to 0.22 μm polyvinylidene fluoride membrane (Merck Millipore). The membrane was blocked with 5% (w/v) non-fat dry milk in tris buffered saline (TBS) for 1 h and subsequently incubated overnight at 4 °C with primary antibodies for STK31(1:500), Flag (1:5000), MCM7, ITGA3, ITGB1, ITGB2, c-Myc, p-c-Myc (Thr244), p-c-Myc (Ser62), FOXP1, CDC7 (1:1000 dilution each) and MCM2, MCM3, MCM4 (1:2000 dilution each),then washed with TBST (0.1% v/v Tween 20 in TBS) and probed for 1 h with an appropriate secondary antibody (1:5000). To confirm equal protein loading, the membranes were subsequently stripped and incubated with antibody against β-actin. Protein bands were visualized by reaction with enhanced chemiluminescence assay kit (Proteintech).

### Kinase activity detection

To confirm that STK31 indeed possesses kinase activity, we used two kits, HTRF KinEASE STK discovery kit (Revvity) and P-Glo Kinase Assay (Promega), to assay the human recombinant STK31 protein (OriGene; NM_031414) according to the manufacturers' instructions. Firstly, kinase activity was measured using a homogeneous time-resolved fluorescence (HTRF) assay. Serially diluted STK31 kinase at various concentrations (with CHK2 as a positive control, OriGene, NM_007194) were incubated with biotinylated STK substrates (S1, S2, and S3), ATP at a final concentration of 100 μmol/L, and 1 × kinase buffer containing 10 mmol/L MgCl_2_ and 1 mmol/L DTT, prior to detection. After incubation, HTRF-based kinase assay was performed on a SpectraMax i3x reader (HTRF mode; excitation 340 nm; emission 620/665 nm, Molecular Devices). ΔRatio was calculated as (Signal_665_/Signal_620_) × 10^4^. For each substrate, ΔRatio vs. kinase concentration data were fitted to a three-parameter log[kinase] vs. response model using GraphPad Prism 10.0. Substrate two was chosen as the optimal substrate based on ΔRatio values, and the kinase concentration–response profile was established. To assess the inhibitory effect of ATO on STK31 kinase activity, the enzymatic reaction was carried out with the addition of ATO (25 μM) or DMSO. The reaction mixtures were incubated at 37 °C for 30 min, after which kinase activity was measured. The P-Glo™ Kinase Assay was also performed to evaluate kinase activity by using a Varioskan Lux under chemiluminescence detection. Relative light units (RLU) were measured and converted to ADP concentrations (μmol/L) based on an ADP standard curve for quantification.

### Co-immunoprecipitation

GBC-SD cells were seeded at a density of 3.5 × 10^6^ cells per culture flask and harvested from flask by gently scraping. The cell pellet was sonicated in 500 μl RIPA buffer with protease inhibitor, followed by centrifugation at 14,000 *g* for 20 min at 4 °C. The protein concentrations of the supernatant were detected by the BCA assay. Five hundred microgram protein aliquots were subjected to Reversible Immunoprecipitation System Kit in accordance with the manufacturer's instructions (Catch and Release v2.0, Merck Millipore). Immunoprecipitation reactions were initiated with equal aliquots of lysates (no-antibody, nonreactive antibody-rabbit IgG (Proteintech) and specific antibody). The no-antibody lysates were directly combined with loading buffer as the input samples. The cell lysates collected from Co-IP kit were combined with loading buffer, and then subjected to heating at 100 °C for 10 min before proceeding to immunoblotting. A horseradish peroxidase-conjugated Protein A was used as the secondary antibody.

### Immunoprecipitation-mass spectrometry (IP-MS) and phosphoproteomics

To identify STK31's direct downstream targets and considering its nature as a serine/threonine kinase, we performed IP-MS and phosphoproteomic analysis using STK31-overexpressing GBC-SD cells. The samples for IP-MS were prepared the same as those for Co-IP. The mass spectrometry was conducted to evaluate the isolated immunoprecipitates. Peptides were solubilized in liquid chromatography mobile phase A and separated using a NanoElute ultra-high performance liquid chromatography (UHPLC) system (solvent A:0.1% formic acid, 2% acetonitrile, solvent B: 0.1% formic acid in acetonitrile). The peptides were separated on the UHPLC system and injected into a Capillary ion source for ionization and then into a timsTOF Pro 2 mass spectrometer for data acquisition.

The samples for phosphoproteomic were collected according to the procedure follows the same steps as Western blot. The peptides were solubilized with liquid chromatography mobile phase A and separated using an EASY-nLC 1200 ultra-high performance liquid chromatography system (solvent A:0.1% formic acid, 2% acetonitrile, solvent B: 0.1% formic acid in acetonitrile). The peptides were injected into the NSI ion source for ionization, and then into the Orbitrap Exploris 480 mass spectrometer for analysis. To increase the effective utilization of the mass spectrum, the automatic gain control (AGC) was set to 1E6 and the maximum injection time was set to Auto.

### Immunofluorescence staining

Cells were fixed with 4% paraformaldehyde for 15 min and permeabilized with 0.5% Triton X-100 (prepared in PBS) at room temperature for 20 min. Next, cells were then blocked with 5% horse serum (Solarbio) for 30 min and incubated with antibodies against STK31(1:500) and c-Myc (1:500) overnight. After the incubation, the cells were stained with anti-Rabbit IgG (H + L) cross-adsorbed secondary antibody (Alexa Fluor 488 conjugated, 1:200, Invitrogen) and anti-mouse IgG(H + L) secondary antibody (Alexa Fluor 594 conjugated, 1:200, ProteinTech). The nuclear counterstain DAPI (Solarbio) was also used. Finally, cell labeled fluorescence was assessed using a Zeiss Confocal Laser Scanning Microscope (LSM 900).

### GBC-SD and NOZ tumor xenograft models

Six-week-old male BALB/c nude mice were supplied by SPF Biotechnology Co., Ltd. Animals were bred and maintained in pathogen-free conditions. All experimental methods in mice were carried out in accordance with the approved guidelines specified by the Animal Experimentation Ethics Committee of the Yan’an Hospital Affiliated to Kunming Medical University (2022020).

### *In vivo* evaluation of tumor inhibition after knocking down of STK31

The subcutaneous xenograft models were employed to study the inhibitory effects *in vivo* after knocking down the expression of STK31 in gallbladder cancer cells GBC-SD and NOZ. Briefly, 2 × 10^6^ NOZ or 1 × 10^6^ GBC-SD cells (shSTK31–05 and shNC) in 100 μl of 7 mg/ml Matrigel (Matrigel Basement Membrane Matrix High Concentration, LDEV-free) were injected subcutaneously into the back of mouse. Tumor growth was measured the length and width every 2 days with a caliper, and tumor volume was calculated using the formula (*a* × *b*^2^)/2, where *a* indicates the length, *b* indicates the width of the tumor. At the same time, each mouse was weighed. The mice were sacrificed by intraperitoneal injection of excess pentobarbital sodium and cervical dislocation on day 24 or 25. The tumor samples were collected for photographing and weighting.

### *In vivo* evaluation of tumor inhibition with ATO treatment

Due to the significant anti-tumor effects of ATO *in vitro* (as shown in result section), further animal studies on ATO were conducted using the subcutaneous gallbladder tumor xenograft-bearing mice. Briefly, 1 × 10^6^ GBC-SD cells in 100 μl of Matrigel were inoculated subcutaneously into the mice on day 0. On day 6, animals were randomized into two groups: vehicle control (0.1% CMC) and ATO treatment group (200 mg/kg). ATO was orally administered daily for 19 days, and tumor volume and body weight of each mouse was measured as same as the previous method. At the end of the treatment period, the mice were anesthetized then sacrificed by cervical dislocation. Then the tumors were weighted before photographing.

### Molecular docking simulation

To identify the potential inhibitors of STK31, we examined the interaction between small molecular compounds from ChEMBL database (https://www.ebi.ac.uk/chembl/) and STK31 using molecular docking method. The structure of STK31(Q9BXU1, https://alphafold.ebi.ac.uk/entry/Q9BXU1) was download from the AlphaFold Protein Structure Database of UniProt. AutoDock Vina software was used for molecular docking, and the ggplot2 package in R language was used for visualization screening results. The binding of ATO and STK31 was simulated by using Pymol software.

### Cellular thermal shift assay (CETSA)

To further confirm the direct regulation of STK31 thermal stabilities by ATO treatment, cellular thermal shift assay experiments were performed as previously described (47). Briefly, GBC-SD cells (or STK31-WT and STK31-ΔKD) were resuspended in ice-cold PBS and lysed by RIPA lysate with cocktail protease and phosphatase inhibitors in ice. The cell lysates were collected by centrifugation (14,000 *g*, 30 min) at 4 °C to remove the debris. The protein concentration was determined by using the BCA protein assay kit (Beyotime). ATO or vehicle control was added to GBC-SD cell lysates (30 μg/12.5 μl) with 50 μM final ATO concentration. The eight aliquots of samples were incubated at 37 °C for 2 h and transferred into 0.2 ml polymerase chain reaction (PCR) tubes. Each sample was heated in parallel for 10 min to the respective temperature (range: 37–72 °C). Subsequently, the samples were centrifuged and supernatant was collected, which was subjected to gel-electrophoresis for Western blot analysis.

### Statistical analysis

Data were expressed as mean ± standard deviation (SD) for *in vitro* studies, and as mean ± standard error of the mean (SEM) for *in vivo* studies. One way analysis of variance (ANOVA) followed by *post hoc* Dunnett’s test were used to compare the treatment groups and the control group. The unpaired Student’s *t* test was performed to compare the difference between two groups. IHC results were analyzed using Chi-square tests. Survival curves were generated using Log-rank tests. Statistical analyses were conducted using a GraphPad Prism 10.1.2 software package (GraphPad Software Inc). Differences were considered to be statistically significant when *p* < 0.05.

## Ethics approval and consent to participate

Ethics approval and consent to participate all procedures involving mice were performed in strict accordance with the Animal Ethics and Welfare Committee (AEWC) of Yan'an Hospital Affiliated to Kunming Medical University (No. 2022020). Title of the approved project: The study on the suppression effect of polyunsaturated fatty acids omega-3 on gallbladder cancer through DNMT1 mediated STK31 methylation. Date of approval: March 16, 2022. The researchers received approval from the Medical Ethics Committee of Yan’an Hospital Affiliated to Kunming Medical University and conducted the study in accordance with the principles outlined in the Declaration of Helsinki (No.2022–026–01). Written informed consent was obtained from all participants. Title of the approved project: The study on the suppression effect of polyunsaturated fatty acids omega-3 on gallbladder cancer through DNMT1 mediated STK31 methylation. Date of approval: March 2, 2022.

## Consent for publication

Written informed consent was obtained from the patient for publication of this case report and accompanying images. A copy of the written consent is available for review by the Editor-in-Chief of this journal on request.

## Data availability

The corresponding authors will make the raw data supporting this study’s findings available upon reasonable request. The tissue microarray expression data have been deposited to the OMIX (https://ngdc.cncb.ac.cn/omix/release/OMIX019407). The mass spectrometry proteomics data have been deposited to the ProteomeXchange Consortium (https://proteomecentral.proteomexchange.org) *via* the iProX partner repository (48, 49) with the dataset identifier PXD082424, PXD082383 and PXD082574.

## Supporting information

This article contains supporting information.

## Conflict of interest

The authors declare that they have no conflicts of interest with the contents of this article.
