# Supplementary material for: STK31 enhances gallbladder cancer development by modulating DNA replication machinery
Source: J Biol Chem. 2026 Sep 8;302(10):113534. doi: 10.1016/j.jbc.2026.113534 (PMC13676983; doi:10.1016/j.jbc.2026.113534)
Supplement: Supplementary file 1 [file mmc1.doc]

**Supplementary Figure 1**

**Supplementary Fig 1.** (A) Epithelial cells in the gallbladder mucosa were detected using CK19 immunofluorescence. (B) The representative immunoblot demonstrated the expression of STK31 in tumor tissue and adjacent normal tissue from GBC patient’s resected sample. (C) Representative images of GB adenocarcinoma specimens and their adjacent normal tissues showing positive and negative staining of STK31 in sections after IHC staining.

**Supplementary Figure 2**

**Supplementary Fig 2.** (A&B) The representative immunoblot demonstrated the knockdown efficiency of STK31 in GBC-SD cells. The histograms showed the quantified results of STK31 levels, which were adjusted with corresponding β-actin protein level and expressed as fold of shNC (mean fold of shNC+SD of three independent experiments). Differences between shNC group and shSTK31 groups were determined by one-way ANOVA followed by *post-hoc* Dunnett’s test, ***P* < 0.01, ****P* < 0.001.

**Supplementary Figure 3**

**Supplementary Fig 3.** The histogram showed quantified results of protein levels in GBC-SD cells, which were adjusted with corresponding β-actin protein level and expressed as folds of control (mean fold of control+S.D. of three independent experiments). Differences between shSTK31 groups and shNC were determined by one way ANOVA followed by *post-hoc* Dunnett’s test, **P*<0.05, ***P*<0.01, ****P*<0.001 as compared with shNC.

**Supplementary Figure 4**

**Supplementary Fig 4.** The histogram showed quantified results of protein levels in NOZ cells, which were adjusted with corresponding β-actin protein level and expressed as folds of control (mean fold of control+S.D. of three independent experiments). Differences between shSTK31 groups and shNC were determined by one way ANOVA followed by *post-hoc* Dunnett’s test, **P*<0.05, ***P*<0.01, ****P*<0.001 as compared with shNC.

**Supplementary Figure 5**

**Supplementary Fig 5.** The morphology of GBC-SD cells with stable knockdown of STK31 under a microscope.

**Supplementary Figure 6**

**Supplementary Fig 6.** (A) Representative immunoblots showed the effects of overexpression on the STK31 protein in GBC-SD. (B) The protein samples used for IP-MS and phosphoproteomics analysis showed a single, distinct band with normal distribution on protein gel staining. (C) Schematic of STK31 WT and mutants used for lentiviral transduction in GBC-SD cells (Created with SnapGene). The full-length STK31 (1–1019 aa) contains a kinase domain (shaded). Δ800–923 lacks residues 800–923; S854D and S854A carry aspartic acid or alanine substitutions at Ser854, respectively (asterisks). Lentiviral constructs were transduced into GBC-SD for functional characterization.

**Supplementary Figure 7**

**Supplementary Fig 7.** (A&B) Cellular thermal shift assay for validation of ATO interacted with STK31-WT or STK31-ΔKD(Δ800-923). Cell lysates were incubated with 50 μM ATO and heated at different temperatures for 10 min, then subjected to immunoblotting. Thermal shift curves of ATO target with increased thermal stability by STK31 immunoblotting in cell lysates. Graph indicates STK31 band intensities quantified using ImageJ. Red curves represent ATO treatment and black curves represent vehicle treatment.（C）The body weight of each mouse was monitored during ATO treatment.
